# A Comparison Between the Complications of Laparoscopic and Open Gastrostomy Tube Insertions: A Single-Center Study From Riyadh, Saudi Arabia

**DOI:** 10.7759/cureus.31992

**Published:** 2022-11-28

**Authors:** Sadeem Atham, Faten Khayat, Deemah Asiri, Nouf Yaqoub, Sarah Alfraih, Yusra S Chachar, Syed F Jamil

**Affiliations:** 1 College of Medicine, King Saud Bin Abdulaziz University for Health Sciences, Riyadh, SAU; 2 College of Science and Health Professions, King Saud Bin Abdulaziz University for Health Sciences, Riyadh, SAU; 3 Research, King Abdullah International Medical Research Center, Riyadh, SAU; 4 College of Medicine, King Abdulaziz University for Health Sciences, Riyadh, SAU; 5 Pedaitrics, King Abdullah Specialized Children's Hospital, Riyadh, SAU

**Keywords:** complications, pediatrics, laparoscopic gastrostomy, open gastrostomy, gastric tube, gastrostomy

## Abstract

Background and objective

Gastrostomy is a procedure that involves placing a feeding tube through the abdominal wall into the stomach to provide nutritional support. There are several modes of gastrostomy tube insertion including laparoscopic-assisted gastrostomy (LAG), percutaneous endoscopic gastrostomy (PEG), and open gastrostomy (OG) procedure, among others. Although it is a widely performed procedure, limited data is available regarding gastrostomy in Saudi Arabia, specifically among the pediatric population. This study aimed to shed more light on different aspects of surgical gastrostomy procedures among pediatric patients at the King Abdullah Specialist Children’s Hospital (KASCH) in Riyadh, Saudi Arabia. The main objective of our study was to report the indications and complications of both LAG and OG insertions in the pediatric population.

Methods

A retrospective cross-sectional study was conducted at KASCH to analyze the different parameters related to LAG and OG insertions, and to evaluate for any association between these modes of insertion and their complications. Pertinent data on children from birth to 14 years of age were collected through consecutive sampling using a chart review. A total of 107 pediatric patients who underwent the procedure from 2016 to 2020 were evaluated.

Results

Demographically, the majority (58%) of gastrostomies were performed in infants (less than a year old). Additionally, our study showed a significantly increased association between LAG and complications such as discharge, (27.12%), skin manifestations (27.12%), and bleeding (10.17%) when compared to OG.

Conclusion

Based on our findings, LAG showed less favorable outcomes in contrast to OG. Further studies should be conducted to validate our findings and ensure consistent results and outcomes among different methods of gastrostomy tube insertion.

## Introduction

Gastrostomy is a method of enteral feeding by the insertion of a tube into an artificial opening through the abdominal wall into the stomach [1]. It is a well-recognized procedure that is often chosen only when other approaches such as enteral supplements, dietary counseling, and appetite stimulation prove to be ineffective. The prevalence of gastric tube insertion increased from 1.9 out of 100 births in 1983-1989 to 3.4 cases out of 100 births between 2000 and 2009 in Perth, Australia [2].

Nutritional support and palliative care are the two main goals of gastrostomy. Patients with debilitating or terminal illnesses, such as those with neurological disorders, including encephalopathy, Guillain-Barre syndrome, repeated strokes, and poliomyelitis, might require gastric tubes to improve their quality of life. Patients with advanced malignancies, severe burns, and oropharyngeal disorders might also need gastrostomies [3]. Moreover, in the pediatric population, gastric tubes are indicated to prevent or correct malnutrition, which is evident in patients receiving chemotherapy, and those with congenital malformations, cystic fibrosis, and oropharyngeal dysmotility [3]. It is worth mentioning that patients with neural disabilities make up the bulk of gastrostomy patients [2]. A study of 314 patients with a gastrostomy tube showed that 75% of them had a neurological impairment, while 13% had an oropharyngeal disorder [4].

The first gastrostomy procedure was performed in 1876 through open surgery. In the following years, non-invasive means were introduced, most notably percutaneous endoscopic gastrostomy (PEG) and laparoscopic-assisted gastrostomy (LAG). These alternative techniques were developed to reduce the overall morbidity and mortality associated with the procedure and to provide different options in terms of cost and degree of invasiveness [1]. Even though it takes longer to perform LAG compared to other techniques, it results in fewer post-procedural complications [5-7]. However, an open gastrostomy (OG) is the preferred method in specific cases where contraindications to other methods of insertion are present [8].

Although gastrostomies are essential and comparatively safe procedures, they are unfortunately associated with complications. Some complications are fairly common and include granuloma formation, wound infection, and gastric outlet obstruction [9]. Granulomas are defined as portions of the skin that undergo inflammation, and they develop around three months postoperatively [9,10]. Granulomas can compromise a suitable area for bacterial invasion, and it could result in bleeding as it is thickly vascularized [11].

The complications mentioned previously were mostly seen in patients older than 14 years of age. As there is insufficient data regarding gastrostomy tube insertions in the general Saudi pediatric population, this study seeks to identify the demographic parameters associated with gastrostomies, common indications, frequency of each gastrostomy tube insertion method, the type of tubes most frequently used, and the association between common complications and methods of insertion in patients from birth up to the age of 14 years at the King Abdullah Specialist Children's Hospital (KASCH) in Riyadh, Saudi Arabia.

## Materials and methods

Study design and setting

A retrospective cohort study was conducted in the Pediatric Gastrointestinal Department at KASCH, King Abdulaziz Medical City, Riyadh, Saudi Arabia. Chart review was utilized to collect the data on age, gender, date of operation, indications, methods of insertion, types of tube used at the time of the surgery, and complications from patient files in the BESTCare data system. The IRB approval was granted on September 7, 2020. Informed consent forms were not required as there was no contact with the patients. However, confidentiality and anonymity were maintained throughout the study.

Identification of study participants

This population-based study included all male and female pediatric patients from birth to 14 years of age who received gastrostomies in the period from January 2016 to December 2020 at KASCH. Those with missing data were excluded. Using non-probability consecutive sampling, the total number of patients was determined to be 107.

Data collection process

Demographic variables including gender and age ranges were collected. Dependent variables collected included the following complications: discharge, tube malfunction, blockage, abdominal distension, accidental removal of the tube, dislodgement, pulling of the tube, bleeding, infections, skin manifestations, intraperitoneal leakage, and adhesions. Independent variables included indications for the procedure, which were failure to thrive (FTT), dysphagia, structural abnormalities, gastroesophageal reflux disease (GERD), and aspiration pneumonia. Methods of tube insertion including OG and LAG were also recorded. Types of tubes used, such as Malecot Tube and Mic-key Button among others, were documented.

Data analysis

Microsoft Excel 2007 was used for data entry, and the data was analyzed using John's Macintosh Project (JMP; JMP Statistical Discovery LLC, Cary, NC). Tables and figures were used to present the results. Frequency and percentage were used for categorical variables. Descriptive analysis was performed to assess age ranges, gender, number of open and laparoscopic gastrostomies performed, type of tube inserted, indications, and complications. Fisher's exact test was used to assess the association between complications and the mode of gastrostomy insertion.

## Results

A total of 107 children underwent surgical gastrostomies in the period from January 2016 to December 2020; 57 (54%) of them were females and 50 (46%) were males. The majority of the patients (63, 58%) were infants (less than a year old). Toddlers (one to two years of age) made up 14% (n=15), preschool-aged children (two to five years of age) made up 15% (n=16), and school-aged children (six years and older) accounted for 12% (n=13) of patients.

The two primary methods of surgical gastrostomy tube placement performed were OG and LAG. The number of participants who underwent each of these procedures was 48 (45%) and 59 (55%), respectively. As for the types of tubes used, the Malecot Gastrostomy Tube was the most frequently used one (81, 75.5%), followed by the Mic-key Button Gastrostomy tube (17, 15.9%). Other tubes such as Bard Wire, Oro-gastrostomy tube, and Red Rubber catheter were used in four (3.7%) patients. The tubes used in five (4.7%) patients were undocumented. With regard to indications, dysphagia, which made up 69%, and FTT, which made up 59%, were more common than GERD and aspiration pneumonia (30%), and structural abnormalities (11%). More details regarding indications are shown in Table 1.

**Table 1:** Indications for gastrostomy tube insertion ^1^Down’s syndrome, MEGDEL syndrome, Sanjad Sakati syndrome, Edward’s syndrome, Aicardi-Goutieres syndrome, Allgrove syndrome, VACTERL syndrome, Zellweger syndrome, Schwartz syndrome ^2^Hypotonia, seizures, hypoxic-ischemic encephalopathy (HIE), stroke, and muscular dystrophy

Indications	N (%)
Failure to thrive: decreased nutritional intake, nutritional deficiency, syndromes^1^, congenital anomalies, nasogastric-tube dependence, malnutrition, chronic wasting, patients requiring nutritional support	63 (59%)
Dysphagia: neurological disorders^2^, global developmental delay, decreased oral intake	74 (69%)
Structural abnormalities: mesenteric artery syndrome hypertrophic, pyloric stenosis, esophageal atresia, esophageal stricture, tracheoesophageal fistula, laryngomalacia, vocal cord paralysis	12 (11%)
Gastroesophageal reflux disease and aspiration pneumonia	32 (30%)

As for complications, the most frequently observed was tube malfunction, which was seen in 22 patients (22%), followed by skin manifestation in 21 (21%), and discharge in 20 (20%) patients. Further details regarding complications are summarized in Table 2.

**Table 2:** Complications of gastrostomy tube insertion

Complications	N (%)
Discharge	20 (20%)
Tube malfunction:	22 (22%)
Loosening	3 (3%)
Breakage	2 (2%)
Leakage	16 (16%)
Wrong placement of the tube	1 (1%)
Blockage	5 (5%)
Abdominal distension	2 (2%)
Accidental removal of the tube	8 (8%)
Dislodgment	2 (2%)
Pulling the tube	5 (5%)
Bleeding	6 (6%)
Infections	5 (5%)
Skin manifestations:	21 (21%)
Granulation tissue	10 (10%)
Excoriations	2 (2%)
Erythema and skin irritation	8 (8%)
Gastrocutaneous fistula	1 (1%)
Intraperitoneal leakage	1 (1%)
Adhesions	1 (1%)

The study found that there was a significant association between laparoscopic gastrostomy tube insertions and complications, particularly discharge (27.12%, p=0.0132), skin manifestations (27.12%, p=0.0305), and bleeding (10.17%, p=0.0230), compared to open gastrostomy tube insertion, which showed no association (p>0.05). Table 3 presents the details regarding associations between complications and methods of insertions.

**Table 3:** Complications related to the method of insertion ^1^Loosening, breakage, leakage, and wrong placement of the tube ^2^Granulation tissue, excoriations, erythema, skin irritation, rash, skin color changes, swelling, and gastrocutaneous fistula

Complications	Method of insertion			
		Laparoscopic-assisted G-tube insertion	Open G-tube insertion	P-value
Discharge		16 (27.12%)	4 (8.33%)	0.0132
Tube malfunction^1^		15 (25.42%)	7 (14.58%)	0.1676
Blockage, abdominal distension		4 (6.78%)	3 (6.25%)	0.9122
Accidental removal of the tube, dislodgment, or pulling the tube		9 (15.25%)	6 (12.50%)	0.6832
Bleeding		6 (10.17%)	0 (0%)	0.0230
Infection		4 (6.78%)	1 (2.08%)	0.2523
Skin manifestations^2^		16 (27.12%)	5 (10.42%)	0.0305
Adhesions		1 (1.69%)	0 (0%)	0.3648
Intraperitoneal leakage		1 (1.69%)	0 (0%)	0.3648

## Discussion

Gastrostomy tubes are the preferred mode of care for patients in need of an alternative method of feeding due to anatomical malformations, swallowing difficulties, and increased need for caloric intake. Several complications have been associated with gastrostomy insertions, and hence choosing the appropriate method of insertion is key to a minimal-risk procedure. Careful assessment of patients' weight, anatomy, and need for general anesthesia or fundoplication prior to the procedure as well as weighing out the complications associated with specific insertions all contribute to the decision-making and, therefore, the outcome of the procedure [12,13].

According to the literature, PEG is usually the preferred method as it is less invasive, requires minimal anesthesia, and is associated with quicker recovery. However, for candidates who are unfit for the percutaneous approach, laparoscopic and open gastrostomies are considered [14]. Our study focused on comparing the outcomes between patients who underwent the laparoscopic approach and those who received open gastrostomies specifically. A significant association was found between LAG and the development of complications, particularly discharge (27.12%, p=0.0132), skin manifestations (27.12%, p=0.0305), and bleeding (10.17%, p=0.0230), compared to OG, which showed no association (p>0.05) in our study; however, the other reported complications had no significant correlation with either method of insertion.

There is a paucity of studies directly comparing LAG and OG in the general pediatric population. Kozlov et al. compared LAG with OG in neonates and infants up to three months of age specifically. Their study showed an overall increased rate of minor complications, such as granulation tissue and peristomal dermatitis, in the OG group [5]. Most of the studies in the literature have compared LAG and OG with other methods such as PEG. In contrast to our study, most studies showed an increased rate of complications with OG. Liu et al. compared all three methods of insertion (OG, LAG, and PEG) and concluded that the overall rate of complications associated with OG was higher (78%, p=0.01) compared to LAG and PEG, which had rates of 44.6% and 54.7%, respectively [6]. Similarly, Sulkowski et al. concluded that patients who underwent OG had poorer outcomes compared to those who underwent other methods; return to emergency departments due to issues related to the GT was observed in 51.7% of patients (p=0.01), granulation tissue was witnessed in 42.1% (p=0.02), and tube dislodgement was more common in this population (37.4%) compared to other groups, which had a combined rate of 8.8% [15]. Furthermore, when comparing LAG with PEG, several studies showed more positive outcomes favoring LAG as it was associated with fewer postoperative complications [16-18]. For instance, one study showed that PEG had a complication rate of 14% compared to LAG, which had a rate of 7.7% [7].

An analysis of data in the literature on this topic showed that OG had higher complication rates compared to other methods of insertion, which contrasts with our findings. It is worth noting that these results were likely influenced by patient-related factors including their age, immune status, major indication for gastrostomy tube insertion, comorbid conditions, and the quality of education provided to the families of the patients [5,19]. Moreover, child activity and discomfort with the gastrostomy tube can cause accidental pulling of the tube leading to skin manifestations due to scratching, which causes erythema, excoriation, and irritation around the site of the stoma. In addition, patients' existing comorbid conditions could predispose them to certain complications; for example, patients with DiGeorge syndrome, a primary immunodeficiency disease, are at higher risk of infections compared to immunocompetent individuals [20]. It is established that congenital heart defects in addition to neurological disorders, whether acute or chronic, can cause malnutrition [21-23]. Lastly, the differences in technical skills required for each of the procedures may also explain the differences in outcome.

This study has a few limitations, including aspects related to our population. Our sample size was relatively small and recruited from only one center. Also, antibiotic prophylaxis prior to gastrostomy insertion was not examined, which is an important predictor of outcomes, especially infections, in this population [24]. Further studies on this topic should be conducted to ensure more consistent results.

## Conclusions

This study focused on pediatric patients who underwent gastrostomy tube insertion, to discuss parameters related to the procedure. Our results showed a correlation between laparoscopic percutaneous gastrostomy insertion and specific complications, in contrast with open gastrostomy. We recommend further studies assessing methods of gastrostomy insertions involving the general pediatric population, as a majority of the studies we found focused either on the adult population or a specific subset of pediatric patients.
